# Intimate partner violence among HIV-positive women in discordant relationships attending care and treatment clinics in Dar es Salaam, Tanzania

**DOI:** 10.3389/fpubh.2022.938624

**Published:** 2023-01-13

**Authors:** Milka Mathania, Nathanael Sirili

**Affiliations:** ^1^Department of Health, Social Welfare and Nutrition, Dar es Salaam City Council, Dar es Salaam, Tanzania; ^2^Department of Development Studies, School of Public Health and Social Sciences, Muhimbili University of Health and Allied Sciences, Dar es Salaam, Tanzania

**Keywords:** HIV-positive, discordant relationship, sero-positive, intimate partner violence, care and treatment clinic

## Abstract

**Background:**

Intimate partner violence (IPV) toward women is a public health concern affecting many countries across the world. Globally, 30% of women experience lifetime physical, sexual, or psychological harm. HIV-positive women in discordant relationships are at higher risk of experiencing IPV than other women. This study aimed to determine the magnitude and factors associated with intimate partner violence among HIV-positive women in discordant relationships attending HIV care and treatment clinics in Dar es Salaam, Tanzania.

**Methods:**

An analytical cross-sectional study was conducted among 411 HIV-positive women who were in discordant relationships attending HIV care and treatment clinics in eight selected healthcare facilities in Dar es Salaam from June 2021 to July 2021. A questionnaire with structured questions on social-demographic information and factors associated with intimate partner violence was used. Data were collected electronically using the Open Data Kit (ODK) system, cleaned, and stored. Descriptive analysis was done and presented in frequency distribution and cross-tabulation. A chi-square was used to determine the differences in variables, and the modified Poisson regression model was used to estimate adjusted prevalence risk ratios (APRRs) with 95% CI for factors associated with IPV. Variables were considered statistically significant at 95% CI and *p* < 0.05. All statistical analyses were conducted using STATA version 15.

**Results:**

The mean age of the participants was 36.2 (standard deviation [SD] ±7.8). The majority of women had attained primary education, i.e., 237 (57.7%). Over 65.7% of HIV-positive women in discordant relationships had ever experienced IPV. Women whose partners had primary and secondary education, were alcohol users, and were solely decision-makers in the family were strongly associated with IPV.

**Conclusion:**

Close to two-thirds of HIV-positive women who were in discordant relationships attending HIV care and treatment clinics in selected healthcare facilities in Dar es Salaam had ever experienced at least one form of IPV. Low level of education of the partner, alcohol use, and power relation in decision-making were significantly associated with IPV. We recommend routine screening of IPV for HIV-positive women who are in discordant relationships attending HIV care and treatment clinics. Joint efforts are needed in addressing the factors associated with IPV in discordant couples.

## Background

Globally, close to one-third of all women in an intimate relationship have ever experienced physical and/or sexual violence from their intimate partner (1). Intimate partner violence (IPV) is any behavior in an intimate relationship that causes physical, sexual, or psychological/emotional harm and controlling toward those in the relationship (2). HIV-positive women are more likely to experience IPV than HIV-negative women (3, 4). Furthermore, HIV-positive women in discordant relationships experience a higher burden of IPV as compared to those in concordant relationships (5). These women are victimized and blamed by their partners for infidelity and the source of problems for the family (6).

Intimate partner violence among HIV-positive women can affect their engagement in HIV care and treatment, lower self-reported antiretroviral therapy (ART) adherence, lead to mental health challenges, lower clinic attendance, and lower odds of viral suppression (7, 8). Furthermore, IPV increases the risk of sexually transmitted infections and HIV transmission to their partners and decreased adherence to ART medication (9, 10). In Africa, 36.5% of women are reported to experience physical or sexual violence at least once in their lifetime (1). In South Africa, HIV-positive women experience more IPV as compared to HIV-negative women (5, 7). Men are reported to be the main perpetrators of IPV. The reasons attributed to men being perpetrators include young age, alcohol abuse, unemployment, and low level of education of men (9, 11). Common factors associated with women's increased likelihood of experiencing violence are young age, low level of education, unemployment, and prior history of sexual abuse (12).

In Tanzania, intimate partner violence against women occurs in all societies irrespective of social, religious, economic, cultural, or ethnic variations. The Tanzania Demographic and Health Survey and Malaria Indicator Survey indicated that women experienced different forms of IPV, with sexual violence being the most common form and is reported to affect 30% of women (13). While it is documented in South Africa that HIV-positive women in discordant relationships are at more risk of experiencing IPV as compared to those in concordant relationships (5); there is a dearth of information regarding this group in Tanzania. Therefore, this study aimed to determine the magnitude and factors associated with intimate partner violence among HIV-positive women in discordant relationships attending HIV care and treatment clinics in Dar es Salaam, Tanzania.

## Materials and methods

### Study design

An analytical cross-sectional study design was conducted among 411 HIV-positive women who were in discordant relationships attending HIV care and treatment clinics in selected healthcare facilities in Dar es Salaam from 21 June 2021 to 11 July 2021. Dar es Salaam is highly populated as compared to other regions in Tanzania (14). It also has the highest number of HIV clients currently attending HIV care and treatment clinics. Women were considered eligible if they were adult HIV positive who were in a discordant relationship and have disclosed their HIV status to their intimate partners.

### Sample size and selection

Using Kish and Leslie formula, a minimum sample size of 400 HIV-positive women was computed. Proportionate sampling was used to obtain a minimum sample to be included from eight HIV care and treatment facilities among 28 public health facilities that have a large number of clients. This refers to the facility with at least more than 2,000 clients currently on ART. From the data collection, a total of 411 HIV-positive women were included in the final sample size (Table 1).

**Table 1:** Proportionate sample size per facility.

**Facility**	**Total no of clients currently attending clinic**	**Sample for each facility**
Amana	6,592	84
Mnazimmoja	6,690	85
Buguruni	4,923	62
Chanika	3,164	11
IDC	4,821	61
Vingunguti	3,008	38
Tabata A	3,310	42
Card Rugambwa	2,192	28
Total	34,700	411

### Data collection methods

The data collection tool was first prepared in English and then translated into Kiswahili, the widely spoken and used language for communication in Tanzania. The questionnaire was adapted from the Domestic Violence Standard Demographic and Health Survey (DHS) tool. For controlling behavior, the questions were adapted from the WHO questionnaire. The questionnaire included structured questions organized into three main themes: (i) the background (social and demographic) information included 7 questions, (ii) the type of IPV experienced involved 13 questions, and (iii) risk factors associated with IPV had 8 questions. Physical violence was considered when a woman answered “yes” to any of the five questions and two questions regarding sexual violence and six questions regarding emotional violence. Controlling behavior was considered when the participant answered “yes” to any of the three questions from the questionnaire.

Before the commencement of the study, the tool was pre-tested among five participants in Kinondoni district at Mwananyamala Regional Referral Hospital, which was deemed fit, as it provided a similar setting as those planned for the study that included a large number of HIV clients currently on ART. The pre-testing aimed at ensuring whether the questions were answering the objectives and they were clear to the participants and carried the intended meaning. No modifications were introduced to the tool, as the tool was found suitable.

### Data analysis

Data were collected electronically using the Open Data Kit (ODK) system (15), cleaned, and stored. Descriptive analysis was done when mean and standard deviation (SD) were computed for continuous variables. Cross-tabulation was used to display the association between independent and dependent variables. The independent variables assessed were those related to the woman's factors such as age, level of education, occupation, and history of prior sexual abuse, partner's factors such as age, education level, occupation, alcohol drinking, and controlling behaviors, and family factors such as type of relationship and decision-maker in the household. We used chi-square in bivariate analysis to determine the association between independent and dependent variables. All variables with a *p*-value <0.2 from the bivariate analysis were added to a multivariable model. We used prevalence risk ratios (PRRs) to determine the factors associated with experiencing intimate partner violence. Since the prevalence of IPV was >10%, we then used a modified Poisson regression model to estimate adjusted prevalence risk ratios (APRRs) with 95% CI for factors associated with IPV. All statistical analyses were conducted using STATA version 15.

### Ethical consideration

Ethical clearance was obtained from the Research and Ethics Committee (REC) of Muhimbili University of Health and Allied Sciences (MUHAS; REF NO DA.282/298/01.C). Permission was requested from Council Medical Officer and then from the Medical Officer in-charge of identified health facilities. For anonymity purposes, the identity of the participants was concealed; therefore, codes instead of names of participants were used. Written informed consent was obtained from eligible women, and the interviews were conducted in private room clinics. Participation in the study was voluntary and no personal identifiers were collected from any study participant for the enhancement of freedom of expression.

## Results

### Socio-demographic characteristics of the study population

A total of 411 HIV-positive women in discordant relationships attending selected eight HIV care and treatment clinics in Dar es Salaam participated in the study. The age of the participants ranged between 30 and 39 with mean age of 36.2 (SD 7.8). More than half, 240 women (58.4%) were in a monogamous marriage. The majority of women had attained primary education, i.e., 237 (57.7%), and nearly half, i.e., 199 (48.4%), were entrepreneurs (Table 2).

**Table 2:** Socio-demographic characteristics of women and their partners (*n* = 411).

**Women**	**Variable**	**Number**	**%**
	**Age group**		
	<25	12	2.9
	25–29	74	18.0
	30–34	100	24.3
	35–39	97	23.6
	**Marital status**		
	Married, monogamy	240	58.4
	Married, polygamy	41	10
	Cohabiting	130	31.6
	**Level of education**		
	Primary	237	57.7
	Ordinary Secondary	157	38.2
	Advanced sec/College	17	4.1
	**Occupation**		
	Employed	74	18
	Self-employed	104	25.3
	Entrepreneur	199	48.4
**Partner**	**Age**		
	Age group		
	<35	24	5.8
	35–39	88	21.4
	40–44	96	23.4
	45–49	106	25.8
	50+	97	23.6
	**Education**		
	Primary	190	46.2
	Ordinary Secondary	160	39.2
	advanced sec/College	60	14.6
	**Occupation**		
	Employed	136	33.1
	Self-employed	188	45.7
	Entrepreneur	79	19.2

### Magnitude and types of intimate partner violence

**Figure 1:**
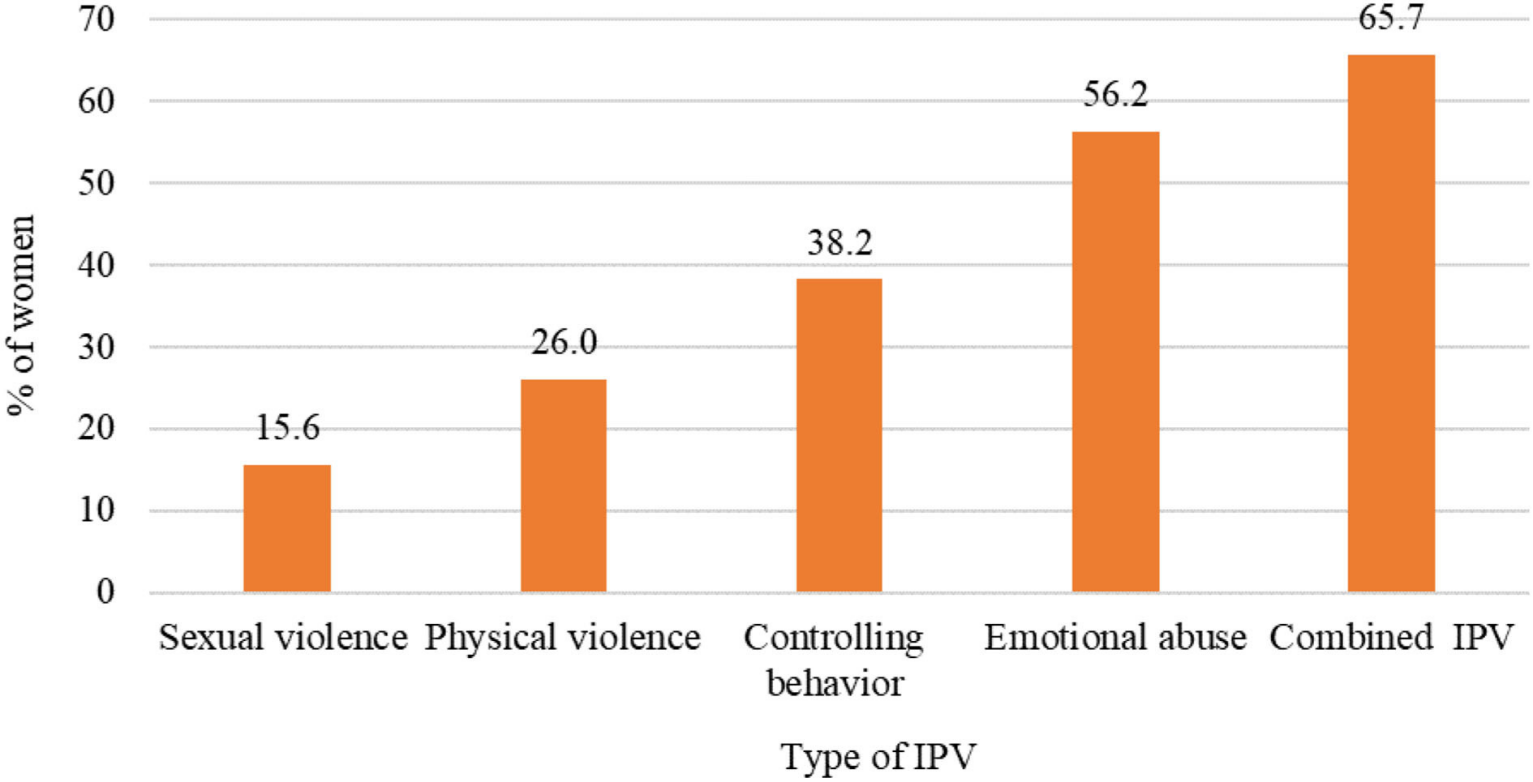
Magnitude and types of intimate partner violence among HIV-positive women in a discordant relationship.

Out of 411 participants, 65.7% had ever experienced one type or a combination of intimate partner violence. Emotional violence was the most experienced form of violence, i.e., 56.2%, controlling behavior was 38.2%, physical violence was 26.0%, and sexual violence was the least, i.e., 15.6%, (Figure 1).

### Factors associated with intimate partner violence

Women aged below 30 years have shown a significant association (*p* < 0.05) risk of being involved in emotional violence.

Women with primary education were strongly associated with physical and sexual violence (*p* < 0.001 and *p* < 0.01), respectively, as compared to those with secondary/college education.

Among different forms of occupation, women who were entrepreneurs had shown a significant association (*p* < 0.001) risk of being involved in physical violence, but those who were employed were shown to have emotional violence at 63.5% as compared to other occupations even though the association was not significant.

History of prior sexual abuse during childhood among HIV-positive women in discordant relationships was strongly associated with all types of intimate partner violence (*p* < 0.001). Furthermore, women with primary education had partners with controlling behaviors as compared to other education levels (*p* < 0.001; Table 3).

**Table 3:** Bivariate analysis showing demographic factors among women associated with intimate partner violence.

**Variable**	***n*** **(%)**	**Physical, *n* (%)**	**Sexual, *n* (%)**	**Emotional, *n* (%)**	**Controlling behaviors, *n* (%)**	**Combined IPV, *n* (%)**
**Age group**						
< 30	86(20.9)	23(26.7)	12(14.0)	59(68.6)^*^	32(37.2)	59(68.6)
30–34	100(24.3)	31(31.0)	9(9.0)	59(59.0)	38(39.0)	59(59.0)
35–39	97(23.6)	20(20.6)	9(9.3)	52(53.6)	31(32.0)	54(55.7)
40–44	63(15.3)	16(25.4)	15(23.8)	35(55.6)	30(47.6)	38(60.3)
45+	65(15.8)	17(26.2)	19(29.2)^**^	26(40.0)	26(40.0)	32(49.2)
**Highest level of education**						
Primary	237(57.7)	90(38.0)^***^	47(19.8)^**^	127(53.6)	110(46.4)^***^	133(56.1)
Secondary/college	174(42.3)	17(10.0)	17(10.0)	104(59.8)	47(27.0)	109(62.6)
**Occupation**						
Employed	74(18.0)	8(10.8)	7(9.5)	47(63.5)	24(32.4)	57(63.5)
Self-employed	104(25.3)	20(19.2)	18(17.3)	55(52.9)	33(31.7)	59(56.7)
Entrepreneur	199(48.4)	69(34.7)^***^	31(15.6)	110(55.3)	82(41.2)!	116(58.3)
Others	34(8.3)	10(29.4)	8(23.5)	19(55.9)	18(52.9)	20(58.8)
**History of prior abuse**						
Yes	31(7.5)	20(64.5)^***^	16(51.6)^***^	25(80.7)^**^	17(54.8)^*^	26(83.9)^**^
No	380(92.5)	87(26.0)	48(12.6)	206(54.2)	140(36.8)	216(56.8)

* P-value < 0.05,

** < 0.01 &

*** < 0.001.

Men aged <35 years were the main perpetrators of emotional and sexual violence (p < 0.001), while those with primary education were involved in physical and sexual violence (p < 0.001 and p < 0.05), respectively.

Male partners with higher education levels were strongly associated with emotional violence (p < 0.001) and overall IPV (p < 0.001).

Employed partners were statistically associated with overall intimate partner violence (p < 0.001) as compared to other types of occupation. Partners who were alcohol drinkers were strongly associated with all forms of intimate partner violence with p < 0.001 (Table 4).

**Table 4:** Bivariate analysis showing demographic factors among partners associated with intimate partner iolence.

**Variable**	***n*** **(%)**	**Physical, *n* (%)**	**Sexual, *n* (%)**	**Emotional, *n* (%)**	**Controlling behaviors, *n* (%)**	**Combined IPV, *n* (%)**
**Age group**						
< 35	24(5.8)	6(25.0)	6(25.0)^*^	17(70.8)^***^	12(50.0)!	18(75.0)!
35-39	88(21.4)	21(23.9)	6(6.8)	59(67.1)	27(30.7)	61(69.3)
40-44	96(23.4)	28(29.2)	11(11.5)	58(60.4)	33(34.4)	67(69.8)
45-49	106(25.8)	29(27.4)	18(17.0)	64(60.4)	50(47.2)	72(67.9)
50+	97(23.6)	23(23.7)	23(23.7)	33(34.0)	35(36.1)	52(53.6)
**Education**						
Primary	190(46.2)	60(31.6)^***^	39(20.5)^*^	75(39.5)	72(37.9)	98(51.6)
Secondary	161(39.2)	44(27.3)	18(11.2)	109(67.7)	69(42.9)!	119(73.9)
College	60(14.6)	3(5.0)	7(11.7)	47(78.3)^***^	16(26.7)	53(88.3)^***^
**Occupation**						
Employed	136(33.1)	28(20.6)	21(15.4)	95(69.9)	48(35.3)	106(77.9)^**^
Self-employed	188(45.7)	51(27.1)	28(14.9)	95(50.5)	72(38.3)	112(59.6)
Entrepreneur	87(21.2)	28(32.2)	15(17.2)	41(47.1)	37(42.5)	52(59.8)

* P-value < 0.05,

** < 0.01 &

*** < 0.001.

Polygamous marriage was significantly associated with the risk of being involved in sexual violence (p < 0.01), though 60.4% of women in monogamy relationships were involved in overall intimate partner violence but were not found to be statistically significant.

The household whereby the partner was the only decision-maker was strongly associated with intimate partner violence and having controlling behaviors (p < 0.05; Table 5).

Women aged 30–34 years were at 30% higher risk of experiencing sexual violence than women in other age groups, i.e., APRR = 0.3, 95% CI (0.1–0.8), *p* < 0.05. Women with primary education had a 1.6 times higher risk of being controlled by their partners as compared to other education levels, i.e., APRR = 1.6, 95% CI (1.1–2.3), *p* < 0.05.

Partners with primary and secondary education were strongly associated with overall IPV perpetration as compared to college education level. Moreover, a partner who was reported to be the solely decision-maker in the family was significantly associated with overall IPV, APRR = 1.9, 95% CI (1.6–2.2), *p* <0.001.

As far as physical violence is concerned, women who reported a history of prior sexual abuse were 1.3 times more likely to have reported physical violence, APRR = 1.3, 95% CI (1.01–1.6), *p* < 0.05 and had 2.6 times higher risk of experiencing sexual violence than those with no such history, APRR = 2.6, 95% CI (1.5–4.4), *p* < 0.01 (Table 5).

**Table 5:** Bivariate analysis showing family demographic factors associated with intimate partner violence.

**Variable**	***n*** **(%)**	**Physical, *n* (%)**	**Sexual, *n* (%)**	**Emotional, *n* (%)**	**Controlling behaviors, *n* (%)**	**Combined IPV, *n* (%)**
**Marital status**						
Married, single wife	240(58.4)	57(23.8)	26(10.8)	139(57.9)	83(34.6)	161(67.1)
Married, polygamy	41(10.0)	13(31.7)	12(29.3)^**^	20(48.8)	21(51.2)!	26(63.4)
Cohabiting	130(31.6)	37(28.5)	26(20.0)	72(55.4)	53(40.8)	83(63.9)
**Decision maker in the household**
Not jointly	155(37.7)	85(54.8)^***^	38(24.5)^***^	129(83.2)^***^	96(61.9)^***^	141(91.0)^***^
Jointly	256(62.3)	22(8.6)	26(10.2)	102(39.8)	61(23.8)	129(50.4)

^*^P-value < 0.05,

** < 0.01 &

*** < 0.001.

## Discussion

We aimed to determine the magnitude and factors associated with intimate partner violence among HIV-positive women in discordant relationships attending HIV care and treatment clinics in Dar es Salaam, Tanzania. About two-thirds of the participants in this study experienced at least one form of IPV. This is significantly higher than IPV in the general population as indicated in the Tanzania Demographic and Health Survey and Malaria Indicator Survey (13), which was 40%. Our findings have also revealed that IPV among HIV-positive women in discordant relationships in Tanzania is higher as compared to our neighboring Uganda where in the same group, IPV was experienced by <45% (7, 16). However, the IPV among HIV-positive women in discordant relationships in Tanzania is far less as compared to the 89.3% that was reported in South Africa (5). Other contextual factors such as increased general criminal records in the population in South Africa as compared to Tanzania may partly explain this variation (17). The magnitude of the overall IPV in the present study could be attributed to the fact that HIV-positive women in discordant relationships are more likely to be victimized by their intimate partners and they are blamed that HIV infection was the result of their infidelity and source of problems in the family (6). This can interfere with their emotional stability and affect their engagement in HIV care and treatment and in economical activities for the wellbeing of their families. Two-thirds is a significantly large proportion and urgent solutions are needed if the goals of an HIV free world are to be attained by 2030. It is high time screening for IPV among all women attending Care and treatment clinic is needed. The latter will help in having real-time data on the magnitude of the problem for policy actions.

Age, history of sexual abuse, level of education of the partner, alcohol abuse, and men as sole decision-makers in the family were the main factors associated with IPV revealed by our study. Our study findings conform to the findings of another study conducted in that and it revealed that at a young age, women are more at risk of experiencing IPV (18). Our study findings are also consistent with what was documented by the WHO multicountry study regarding women's young age and association with experiencing IPV (12). In Tanzania, as per many socio-cultural practices, many instances of IPV go unreported and for that matter, tailored strategies need to be instituted to start with at the care and treatment clinic (CTC) so that identification and addressing of IPV can be done across the different age groups attending CTC.

The history of prior sexual abuse as revealed in our study is in line with what was documented in Nigeria and a previous study in Tanzania (19, 20). Our study results explain the effects of childhood sexual abuse. Adult survivors of sexual abuse may be less skilled at self-protection. They are more apt to accept and to relate to being victimized by others, the tendency of being victimized repeatedly may be the result of a general vulnerability in dangerous situations and exploitation by untrustworthy people (21).

As for other previous studies, our study findings have shown a strong correlation between IPV and education level of partners (22). In our study, partners with primary and secondary education levels were more likely to engage in IPV as compared to partners with college education levels. Other studies indicated that incidence of IPV decreased with higher education level of partners (12, 18). We feel that investing in HIV education and IPV as health promotion campaigns may be a good starting point in addressing the IPV.

In this study, partners who were sole decision-makers in the household had a 1.9 times higher chance of perpetrating IPV than those who jointly make the decision for their families. These findings, which suggested that women who had a decision-making autonomy had a lower likelihood of experiencing IPV, confirm the results from Nigeria (23). Women in our communities tend to accept a submissive attitude and hence cannot decide for themselves and their families, this increases the risk of experiencing IPV (24).

### Study limitations

In this study we had a couple of limitations, the interview was done face to face, so this could have led to social desirability and recall bias, but their responses were regarded as fairly correct. We also interviewed only women and hence this study relied on women's reports of their partner's characteristics, which is also subject to bias. Therefore, we mitigated this by including a large sample size in the study. Furthermore, the study was cross-sectional, which made it difficult to determine causal inference.

### Conclusions and recommendations

This study found high intimate partner violence among HIV-positive women in discordant relationships. HIV care and treatment clinics should strengthen routine IPV screening, especially for women whose partners are alcohol drinkers, with primary and secondary education levels and those reported to be the only decision-makers in the family. These women should be screened and identified during individual counseling and proper efforts should be taken to respond to their needs. Partners should also be encouraged to accompany their spouses to the clinic for more health education pertaining to HIV prevention and the effects of IPV perpetration. Health facilities should strengthen IPV record keeping and documentation for planning and further research activities. Policies and laws must be well adhered to and strict measured should be taken against the IPV perpetrators. Further studies are recommended on qualitative study for more information on factors associated with IPV and conducting large scale studies involving partners.

## Data availability statement

The original contributions presented in the study are included in the article/supplementary material, further inquiries can be directed to the corresponding author.

## Ethics statement

The studies involving human participants were reviewed and approved by Research and Ethics Committee (REC) of Muhimbili University of Health and Allied Sciences (MUHAS). The patients/participants provided their written informed consent to participate in this study.

## Author contributions

MM conceptualized the idea and collected data for the study. NS co-concentualized and finetuned the study. MM and NS analyzed the data, wrote the manuscript, and revised the manuscript. All authors approved the final manuscript.
